# A hypothesis on the aetiology of adenomyosis: is there an association between cumulative number of menstruations and severity of adenomyosis on ultrasound?

**DOI:** 10.1016/j.eurox.2026.100472

**Published:** 2026-06-24

**Authors:** Eva J.E. de Bock, Cilla Verbeek, Eva Klinkenberg, Jos W.R. Twisk, Nicole B. Burger, Lynda J.M. Juffermans, Judith A.F. Huirne, Robert A. de Leeuw

**Affiliations:** a Department of Obstetrics and Gynaecology, Amsterdam University Medical Centre, Amsterdam, the Netherlands; b Amsterdam Reproduction and Development, Amsterdam University Medical Centre, Amsterdam, the Netherlands; c Department of Epidemiology and Data Science, Amsterdam University Medical Centre, Amsterdam, the Netherlands

**Keywords:** Combined oral contraceptives, Microtrauma, Myometrium, Ultrasonography, Uterus

## Abstract

**Rationale:**

Adenomyosis is a common uterine disorder, yet its aetiology remains debated. The tissue injury and repair (TIAR) hypothesis suggests that repeated microtrauma from menstrual contractions damages the endometrial-myometrial interface, thereby contributing to development and progression of adenomyosis. We investigated whether cumulative menstrual exposure is associated with severity of adenomyosis on ultrasound.

**Methods:**

This exploratory retrospective study was conducted at Amsterdam University Medical Centre, as a sub-analysis of the prospective *UteroVue* cohort study. For the *UteroVue* study, premenopausal participants with and without adenomyosis were recruited via the benign gynaecology outpatient clinic and through flyering, and underwent transvaginal ultrasound. The percentage of myometrium affected by Morphological Uterus Sonographic Assessment features of adenomyosis was estimated. For this sub-analysis, cumulative menstrual exposure was derived from menstrual history questionnaires, accounting for pregnancy, lactation, and hormonal menstruation suppression. The association between cumulative menstruations and disease severity was assessed using multivariable regression analyses with collinearity evaluation.

**Results:**

We included 119 participants in the final analysis. The median age of the study population was 27 years, of whom 79% were nulliparous and 14% had a history of uterine surgery. Previous hormonal menstruation suppression with the oral contraceptive pill was reported by 87% of participants, with a median duration of use of 7 years. In addition, 32% had previously used a hormonal intrauterine device, with a median duration of use of 3 years. Age was excluded from multivariable analyses due to collinearity (r = 0.72). After adjustment for vaginal births and uterine surgeries, cumulative menstrual exposure was associated with severity of adenomyosis (β=0.003; 95% CI 1.001–1.006; p < 0.05).

**Conclusion:**

Higher cumulative menstrual exposure was associated with increased severity of adenomyosis, supporting the TIAR hypothesis. Given the retrospective study design and collinearity with age, results should be interpreted with caution.

## Highlights

• Higher cumulative menstruations are associated with more severe adenomyosis
• Findings support the tissue injury and repair hypothesis of adenomyosis
• Association is independent of vaginal birth and uterine surgeries

## Introduction

Few gynaecological conditions remain as mysterious as adenomyosis. This uterine disorder is characterised by endometrial glands and stroma within the myometrium and is associated with dysmenorrhea, heavy menstrual bleeding, and subfertility [1]. These symptoms can be profoundly disruptive to daily life [2].

Beyond this knowledge, much about the condition remains unknown. This is reflected in the wide variation in reported prevalence, ranging from 7% to 67% depending on study population and diagnostic modality (ultrasound, magnetic resonance imaging, or histopathology) [3], [4], [5], [6], [7], [8], [9], [10], [11]. The lack of effective treatments further illustrates these knowledge gaps: hysterectomy is unsuitable for those desiring pregnancy, and hormonal therapies often fail to provide adequate symptom relief [12]. To develop effective treatment and prevention strategies, it is essential to uncover the underlying aetiology.

Two main hypotheses have been proposed: the “metaplasia” and “invagination” theories [13]. The latter postulates that disruption of the endo-myometrial interface enables endometrial invasion into the myometrium. Initially, the invagination theory focused on macrotrauma, such as vaginal birth and surgeries. However, the “tissue injury and repair” (TIAR) hypothesis posits that, in addition to macrotrauma, repeated microtrauma from physiological uterine menstrual contractions damages the endo-myometrial interface [14]. This microtrauma triggers a chronic injury-repair cycle, characterised by inflammation, local oestrogen production and enhanced uterine contractility. The resulting vicious cycle weakens the endo-myometrial interface and induces cellular changes in basal endometrial cells, increasing their proliferation, migratory capacity, and resistance to apoptosis. This facilitates endometrial invasion [14].

Based on the TIAR hypothesis, a higher cumulative number of menstruations may cause more microtrauma, possibly resulting in more extensive adenomyosis. Supporting this, studies have shown that early menarche and short menstrual cycles are associated with adenomyosis [15], [16]. Nevertheless, the TIAR hypothesis remains debated. Therefore, we explored whether the cumulative number of menstruations is associated with the severity of adenomyosis on ultrasound.

## Methods

This exploratory retrospective cohort study was conducted at Amsterdam University Medical Centre, a tertiary referral centre for adenomyosis, as a sub-analysis of the prospective *UteroVue* cohort study. The study was approved by the institutional review board (NL83391.018.23).

### Participants

Participants for the *UteroVue* study were recruited via the benign gynaecology outpatient clinic and via flyering. Inclusion criteria were premenopausal participants aged ≥ 18 years with a uterus in situ. Exclusion criteria were current pregnancy and intrauterine device use. For this sub-analysis, additional exclusion criteria were a dominant uterine disorder other than adenomyosis, incomplete or invalid questionnaire data, and adenomyosis in the outer third of the myometrium presumed secondary to endometriosis.

### Study procedure

During the study consultation, clinical information was obtained, including current medication use, obstetric and surgical history, and current gynaecological symptoms. All participants subsequently underwent a transvaginal ultrasound examination using a HERA W10 machine with an EV2–10A transvaginal probe (Samsung Medison, Seoul, Republic of South Korea). After the study visit, two experienced sonographers (E.B. and N.B.) assessed the 2D and 3D B-mode and power Doppler scans by consensus, blinded to clinical data. The scans were evaluated on the presence of direct and indirect Morphological Uterus Sonographic Assessment (MUSA) features of adenomyosis [17]. Previously, the MUSA group proposed a classification of disease severity based on the estimated percentage of myometrium affected by MUSA features of adenomyosis, categorising adenomyosis as mild (<25% affected myometrium), moderate (25–50% affected myometrium), or severe (>50% affected myometrium) [18], [19]. In the present study, the severity of adenomyosis was recorded as a continuous estimate of the percentage of affected myometrium.

### Menstrual history

**Fig. 1:**
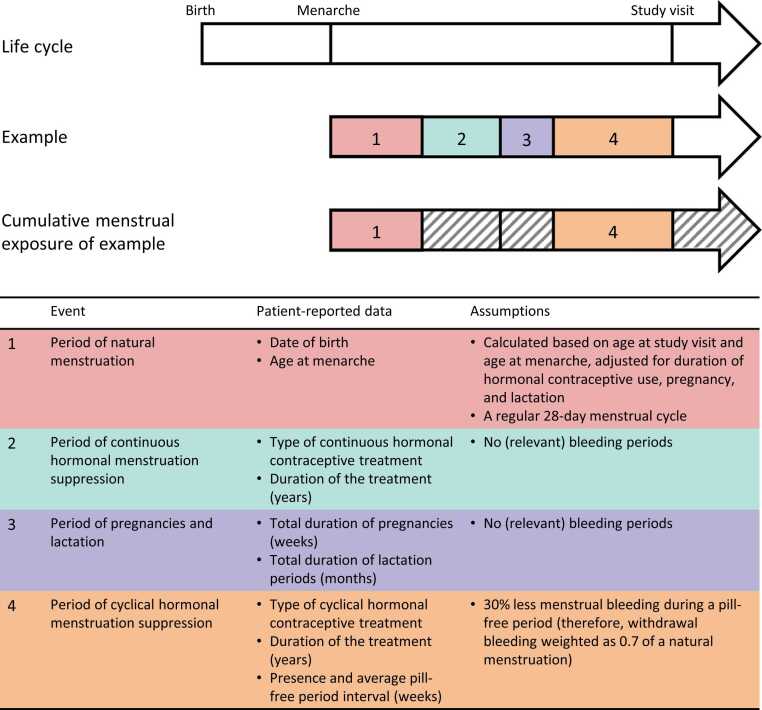
Example of cumulative menstrual exposure estimation.

For this retrospective sub-analysis, participants were invited to complete a questionnaire about their menstrual history after their study visit. They reported age at menarche; cumulative weeks of pregnancy and breastfeeding; use and duration of hormonal menstruation suppression, including pill-free intervals; and a history of endometritis, pelvic inflammatory disease, chlamydia, and gonorrhoea, all retrospectively up to the time of the study visit. Data were verified after collection, and participants were contacted by telephone in case of discrepancies (e.g. reported medication use with a duration of 0 years). If participants could not be reached, a duration of 0.4 years was imputed, assuming that durations < 0.5 years may have been rounded down to zero. The cumulative number of menstruations was estimated accounting for pregnancy, lactation, and hormonal menstruation suppression use (Fig. 1). Withdrawal bleeding during cyclical hormonal menstruation suppression (oral contraceptive pill, vaginal ring, contraceptive patch) was weighted at 0.7 of a natural menstruation to account for the approximately 30% less vaginal blood loss compared to menstruation [20]. Continuous hormonal menstruation suppression (hormonal intrauterine device, progestogen-only pill, contraceptive injection, contraceptive implant) was assumed to result in amenorrhoea.

### Statistical analyses

As this was an exploratory study, no formal sample size calculation was performed. Statistical analyses were performed using IBM SPSS 28.0, with *p*-value < 0.05 considered statistically significant. If the outcome variable was skewed, a logarithmic transformation was applied; in such cases, a constant of 1 was added to all observations to accommodate zero values. The primary study endpoint was the association between cumulative menstruations and severity of adenomyosis. To assess this association, linear regression analyses were used. Based on the literature, potential confounding variables were identified and evaluated: vaginal births, prior uterine surgeries (caesarean sections, myomectomies, curettages, endometrial ablations), and age [7], [15], [21], [22]. Confounders were retained if their inclusion resulted in a ≥ 10% change in the regression coefficient [23]. Potential multicollinearity was assessed using correlation analyses; variables showing collinearity (*r* > 0.7) were excluded. Subsequently, a multivariable linear regression analysis was performed, including cumulative menstruations and the selected confounders. Independent associations of each confounder with severity of adenomyosis were assessed within the same model.

## Results

A total of 264 participants provided informed consent between 2024 and 2025. After excluding participants due to dominant uterine fibroids (n = 91), malignancy (n = 4), PEComa (n = 1), uterine necrosis (n = 1), incomplete questionnaires (n = 45), implausible data resulting in a negative number of estimated menstruations (n = 2), adenomyosis presumed secondary to endometriosis (n = 1), the analysis included 119 participants. Among the included participants, 13% had endometriosis (n = 16). Of the participants with endometriosis, 38% had mild adenomyosis, 38% moderate adenomyosis, and 25% severe adenomyosis. Their general characteristics and hormonal menstruation suppression medication use are shown in Table 1.

**Table 1:** General characteristics and hormonal menstruation suppression medication use of the participants.

Characteristic	Value, N = 119
Age^a^ (years)	27.0 (24.0 – 34.0)
Body Mass Index^a^ (kg/m^2^)	23.5 (21.1 – 26.7)
Ancestral origin^b^	
*Africa*	5 (4%)
*Asia*	3 (3%)
*Europe*	98 (82%)
*South America*	4 (3%)
*Mixed*	9 (8%)
Age at menarche^c^ (years)	12.7 ± 1.5
Gravidity^b^	
*0*	86 (72%)
*1*	10 (8%)
*≥2*	23 (19%)
Parity^b^	
*0*	94 (79%)
*1*	9 (8%)
*≥2*	16 (13%)
Prior gynaecological inflammatory conditions^b^	
*Chlamydia*	17 (14%)
*Pelvic inflammatory disease*	1 (1%)
*Endometritis*	15 (13%)
Endometriosis	16 (13%)
Prior uterine surgery^b^	17 (14%)
*Caesarean section*	4 (3%)
*Hysteroscopic myomectomy*	1 (1%)
*Curettage*	10 (8%)
*Endometrial ablation*	1 (1%)
Menstrual cycle phase at time of study visit^b^	
*Pre-ovulatory*	36 (30%)
*Post-ovulatory*	25 (21%)
*Suppressed menstrual cycle*	58 (49%)
Gynaecological symptoms^b^	
*No symptoms*	95 (80%)
*Heavy menstrual bleeding*	7 (6%)
*Dysmenorrhea*	10 (8%)
*Chronic pelvic pain*	13 (11%)
*Deep dyspareunia*	9 (8%)
*Subfertility*	1 (1%)
Cyclical hormonal menstruation suppression *Oral contraceptive pill*^b^	103 (87%)
*Duration in years*^a^	7.0 (3.0-11.0)
*Vaginal ring*^b^	9 (8%)
*Duration in years*^a^	1.0 (0.7-1.0)
*Contraceptive patch*^b^	2 (2%)
*Duration in years*^a^	1.3 (1.0-1.3)
Continuous hormonal menstruation suppression	
*Hormonal intrauterine device*^b^	38 (32%)
*Duration in years*^a^	3.0 (1.0-5.0)
*Progestogen-only pill*^b^	24 (20%)
*Duration in years*^a^	1.0 (0.1-1.0)
*Contraceptive injection*^b^	12 (10%)
*Duration in years*^a^	1.0 (0.5-1.8)
*Contraceptive implant*^b^	10 (8%)
*Duration in years*^a^	1.0 (0.8-3.6)
Estimated myometrium affected by adenomyosis	
*No adenomyosis (0%)*	10 (8%)
*Mild adenomyosis (1-24%)*	74 (62%)
*Moderate adenomyosis (25-50%)*	20 (17%)
*Severe adenomyosis (>50%)*	15 (13%)
Direct MUSA features of adenomyosis	
*Subendometrial lines and/or buds*	102 (86%)
*Myometrial cysts*	36 (30%)
*Hyper-echoic islands*	76 (64%)
Indirect MUSA features of adenomyosis	
*Fan-shaped shadowing*	54 (45%)
*Globular uterus*	10 (8%)
*Asymmetrical thickening*	21 (18%)
*Irregular junctional zone*	98 (82%)
*Interrupted junctional zone*	58 (49%)

a Median (25^th^ percentile - 75^th^ percentile).

b Count of participants (percentage).

c Mean ± standard deviation. MUSA = Morphological Uterus Sonographic Assessment.

In the unadjusted analyses, a statistically significant association was observed between the cumulative number of menstruations and severity of adenomyosis (log-transformed *β* = 0.005 on the ln(*percentage affected myometrium*+1) scale, Exp(*β*) = 1.005, 95% confidence interval (CI)= 1.002–1.007, *p* < 0.01). Age showed a moderate correlation with the cumulative number of menstruations (*r* = 0.72), indicating collinearity; therefore, age was not included in adjusted models. After adjustment for number of vaginal births and uterine surgeries, included as confounders due to ≥ 10% change in the regression coefficient for cumulative menstrual exposure, the association between cumulative number of menstruations and severity of adenomyosis remained statistically significant (log-transformed *β*=0.003 on the ln(*percentage affected myometrium*+1) scale*,* Exp(*β*)= 1.003, 95% CI= 1.001–1.006, *p* < 0.05). In addition, the confounders included in the model, vaginal births and uterine surgeries, were also independently associated with severity of adenomyosis (both *p* < 0.05).

## Discussion

Understanding the aetiology of adenomyosis is crucial for advancing treatment and prevention approaches. In this study, we investigated the TIAR hypothesis by examining the relationship between menstrual exposure and the severity of adenomyosis on ultrasound. We found that the cumulative number of menstruations was significantly associated with disease severity. Specifically, each menstruation corresponded to a 0.3% increase in affected myometrium, indicating a clinically relevant impact over a lifetime, considering an average of approximately 450 menstruations [24], [25]. These findings support the hypothesis that repeated microtrauma to the endo-myometrial interface during menstruation enables endometrial invasion, driving the development of adenomyosis.

This is the first empirical study on the relationship between menstrual exposure and adenomyosis. Thereby, it provides an important contribution to the largely theoretical literature on TIAR [26]. Furthermore, our findings support the invagination theory on which the TIAR model builds, as uterine surgeries and vaginal birth were both associated with adenomyosis. This is in line with previous studies [15], [27], [28], [29].

Several limitations should be considered. First, menstrual history data were collected retrospectively, which may have introduced recall bias. Second, selection bias might have occurred because only participants who completed the menstrual history questionnaire were included. This might have resulted in underrepresentation of participants with complex medication histories, who might have found it difficult to report their extensive medication history. Third, age was moderately correlated with cumulative menstrual exposure, hindering our ability to assess their independent effects. These variables could not be separated, as the limited sample size prevented subgroup analyses of similarly aged participants with varying menstrual exposures. Fourth, the relatively young age of the study population may limit generalisability. Fifth, the weighting factor applied to withdrawal bleeding contributed to imprecision in menstrual exposure estimation. Given these limitations, our findings should be interpreted with caution. Nonetheless, such limitations are expected in an initial exploration of the understudied TIAR hypothesis.

The potential impact of menstrual exposure on progression of adenomyosis is increasingly relevant, as lifetime menstrual exposure has risen substantially over time. In industrialised countries today, the lifetime number of ovulatory menstruations is roughly three to ten times higher than in the 18th century, due to fewer pregnancies, shorter durations of lactational amenorrhea, earlier menarche, and later menopause [24], [25]. If the TIAR hypothesis is correct, this would imply that the lifetime risk of severe adenomyosis in these countries is higher nowadays as a consequence of increased menstrual exposure. Under this assumption, it may be worthwhile to explore whether hormonal menstruation suppression could prevent progression of adenomyosis.

To disentangle the effects of age and menstrual exposure, further research with larger populations with broad variation in cumulative menstrual exposure within age groups is required to enable age-based subgroup analyses. Furthermore, prospective studies with serial ultrasound exams are necessary to observe disease progression over time. Lastly, assessment of disease severity may benefit from incorporating additional tissue characteristics into the ultrasound exam, such as microcirculation and tissue stiffness, through the use of contrast-enhanced ultrasound and elastography [30], [31].

## Conclusion

Our findings suggest that cumulative menstrual exposure is associated with severity of adenomyosis, supporting the TIAR hypothesis. However, causality cannot be inferred and the role of age remains uncertain. Further prospective studies are needed to clarify the relationship between menstrual exposure, age, and severity of adenomyosis.

## CRediT authorship contribution statement

**Eva J.E. de Bock:** Writing – review & editing, Writing – original draft, Visualization, Project administration, Methodology, Investigation, Formal analysis, Data curation, Conceptualization. **Cilla Verbeek:** Writing – review & editing, Writing – original draft, Visualization, Methodology, Investigation, Formal analysis, Data curation, Conceptualization. **Eva Klinkenberg:** Methodology, Investigation, Formal analysis, Data curation. **Jos W.R. Twisk:** Methodology. **Nicole B. Burger:** Writing – review & editing, Supervision, Methodology, Conceptualization. **Lynda J.M. Juffermans:** Writing – review & editing, Supervision, Methodology, Funding acquisition, Conceptualization. **Judith A.F. Huirne:** Writing – review & editing, Supervision, Methodology, Funding acquisition, Conceptualization. **Robert A. de Leeuw:** Writing – review & editing, Supervision, Methodology, Conceptualization.

## Declaration of Competing Interest

The authors have nothing to declare.

## References

[1] Naftalin J., Hoo W., Nunes N., Holland T., Mavrelos D., Jurkovic D. (2016). Association between ultrasound features of adenomyosis and severity of menstrual pain. Ultrasound Obstet Gynecol.

[2] Nelsen L.M., Lenderking W.R., Pokrzywinski R., Balantac Z., Black L., Pokras S. (2018). Experience of Symptoms and Disease Impact in Patients with Adenomyosis. Patient.

[3] Naftalin J., Hoo W., Pateman K., Mavrelos D., Holland T., Jurkovic D. (2012). How common is adenomyosis? A prospective study of prevalence using transvaginal ultrasound in a gynaecology clinic. Hum Reprod.

[4] Pinzauti S., Lazzeri L., Tosti C., Centini G., Orlandini C., Luisi S. (2015). Transvaginal sonographic features of diffuse adenomyosis in 18-30-year-old nulligravid women without endometriosis: association with symptoms. Ultrasound Obst Gyn.

[5] Yeniel O., Cirpan T., Ulukus M., Ozbal A., Gundem G., Onener S. (2007). Adenomyosis: prevalence, risk factors, symptoms and clinical finding's. Clin Exp Obstet Gyn.

[6] Bergholt T., Eriksen L., Berendt N., Jacobsen M., Hertz J.B. (2001). Prevalence and risk factors of adenomyosis at hysterectomy. Hum Reprod.

[7] Vercellini P., Viganò P., Somigliana E., Daguati R., Abbiati A., Fedele L. (2006). Adenomyosis:: epidemiological factors. Best Pract Res Cl Ob.

[8] Pervez S.N., Javed K. (2013). Adenomyosis among samples from hysterectomy due to abnormal uterine bleeding. J Ayub Med Coll Abbottabad.

[9] Kissler S., Zangos S., Kohl J., Wiegratz I., Rody A., Gätje R. (2008). Duration of dysmenorrhoea and extent of adenomyosis visualised by magnetic resonance imaging. Eur J Obstet Gyn R B.

[10] Abu Hashim H., Elaraby S., Fouda A.A., El Rakhawy M. (2020). The prevalence of adenomyosis in an infertile population: a cross-sectional study. Reprod Biomed Online.

[11] Puente J.M., Fabris A., Patel J., Patel A., Cerrillo M., Requena A. (2016). Adenomyosis in infertile women: prevalence and the role of 3D ultrasound as a marker of severity of the disease. Reprod Biol Endocrinol.

[12] Etrusco A., Barra F., Chiantera V., Ferrero S., Bogliolo S., Evangelisti G. (2023). Current medical therapy for adenomyosis: from bench to bedside. Drugs.

[13] García-Solares J., Donnez J., Donnez O., Dolmans M.M. (2018). Pathogenesis of uterine adenomyosis: invagination or metaplasia?. Fertil Steril.

[14] Leyendecker G., Wildt L. (2011). A new concept of endometriosis and adenomyosis: tissue injury and repair (TIAR). Horm Mol Biol Clin I.

[15] Parazzini F., Vercellini P., Panazza S., Chatenoud L., Oldani S., Crosignani P.G. (1997). Risk factors for adenomyosis. Hum Reprod.

[16] Templeman C., Marshall S.F., Ursin G., Horn-Ross P.L., Clarke C.A., Allen M. (2008). Adenomyosis and endometriosis in the California Teachers Study. Fertil Steril.

[17] Trommelen L.M., De Leeuw R.A., Van den Bosch T., Huirne J.A.F. (2024). Grading sonographic severity of adenomyosis: a pilot study assessing feasibility and interobserver reliability. J Ultrasound Med.

[18] Van den Bosch T., de Bruijn A.M., de Leeuw R.A., Dueholm M., Exacoustos C., Valentin L. (2019). Sonographic classification and reporting system for diagnosing adenomyosis. Ultrasound Obst Gyn.

[19] De Bock E.J.E., De Leeuw R.A., Huirne J.A.F., Burger N.B., Juffermans L. 2025. Consens Défin a Healthy Uterus Results a Modif Delphi Proced.

[20] Hefnawi F., Younis N., Zaki K., Rassid S.A., Mekkawi T. (1970). Menstrual bleeding with the pill and the IUD. IPPF Med Bull.

[21] Vannuccini S., Petraglia F. (2019). Recent advances in understanding and managing adenomyosis. F1000Res.

[22] Leyendecker G., Wildt L., Mall G. (2009). The pathophysiology of endometriosis and adenomyosis: tissue injury and repair. Arch Gynecol Obstet.

[23] (2000). FRANK KA. Impact of a Confounding Variable on a Regression Coefficient. Sociol Methods & Res.

[24] Eaton S.B., Pike M.C., Short R.V., Lee N.C., Trussell J., Hatcher R.A. (1994). Women's reproductive cancers in evolutionary context. Q Rev Biol.

[25] Vercellini P., Bandini V., Viganò P., Di Stefano G., Merli C.E.M., Somigliana E. (2024). Proposal for targeted, neo-evolutionary-oriented, secondary prevention of early-onset endometriosis and adenomyosis. Part I: pathogenic aspects. Hum Reprod.

[26] Habiba M., Gordts S., Bazot M., Brosens I., Benagiano G. (2020). Exploring the challenges for a new classification of adenomyosis. Reprod Biomed Online.

[27] Panganamamula U.R., Harmanli O.H., Isik-Akbay E.F., Grotegut C.A., Dandolu V., Gaughan J.P. (2004). Is prior uterine surgery a risk factor for adenomyosis?. Obstet Gynecol.

[28] Elsherbini M., Koga K., Hiraoka T., Kumasawa K., Maki E., Satake E. (2022). Establishment of a novel mouse model of adenomyosis suitable for longitudinal and quantitative analysis and perinatal outcome studies. Sci Rep-Uk.

[29] Hao M.H., Liu X.S., Guo S.W. (2020). Adenomyosis in mice resulting from mechanically or thermally induced endometrial-myometrial interface disruption and its possible prevention. Reprod Biomed Online.

[30] De Bock E.J.E., Stoelinga B., Van Waesberghe J.H.T.M., Kandi F.I., Dinis Fernandes C., Mischi M. (2025). Uterine Contrast-Enhanced Ultrasound (CEUS): new insights into adenomyosis using an innovative technique. WFUMB Ultrasound Open.

[31] Stoelinga B., Hehenkamp W.J.K., Brölmann H.A.M., Huirne J.A.F. (2014). Real-time elastography for assessment of uterine disorders. Ultrasound Obst Gyn.

